# Acromegaly Initially Presenting with Severe Infectious Diseases: A Case Report

**DOI:** 10.31662/jmaj.2021-0150

**Published:** 2021-12-24

**Authors:** Eriko Tani, Tomonori Hirashima, Takamasa Hasegawa, Daisuke Aohara, Yuri Oshima, Yusuke Sakurai, Kaho Hirai, Naoki Yoshimoto, Mana Nishida, Yu Tateishi, Kenichi Minami

**Affiliations:** 1 Department of Respiratory Medicine, Ishikiri-Seiki Hospital, Higashi-Osaka, Japan; 2 Department of Thoracic Oncology, Ishikiri-Seiki Hospital, Higashi-Osaka, Japan; 3 Department of Endocrinology, Ishikiri-Seiki Hospital, Higashi-Osaka, Japan; 4 Department of Nephrology, Ishikiri-Seiki Hospital, Higashi-Osaka, Japan

**Keywords:** acromegaly, early diagnosis, septic pulmonary embolism, diabetes mellitus, diabetic ketoacidosis

## Abstract

A 39-year-old man presented with worsening fever, cough, and fatigue. He was immediately admitted to the intensive care unit (ICU) and was found to have sepsis, septic pulmonary embolism, right empyema, liver abscess, pyelonephritis, and a prostate abscess, with background diabetes mellitus. While receiving treatment, an ICU nurse noticed that the patient’s toe tips were too large to fit the clamp device of pulse oximeters. Thus, we re-examined the patient and confirmed that he had clinical features indicative of acromegaly including bulging eyebrows, enlarged nose and lips, large feet, and prognathism. He and his family had not noticed these features except for his enlarged feet. We evaluated the patient further for acromegaly, and a pituitary mass was detected via contrast-enhanced head magnetic resonance imaging. Whole-body computed tomography also revealed thickened heel pads, cauliflower deformity, frontal sinus enlargement, sella turcica enlargement, and mandibular malocclusion. A 75 g oral glucose tolerance test was performed to investigate abnormal secretion of growth hormone (GH), and the results revealed a paradoxical increase in GH levels. The patient was then diagnosed with acromegaly according to the clinical guidance of the Japan Endocrine Society. Acromegaly develops slowly; thus, to improve patients’ prognoses, physicians including internists, family physicians, and endocrinologists should include acromegaly in their differential when signs are apparent.

## Introduction

Acromegaly is a rare chronic disorder that is commonly caused by excessive growth hormone (GH) secretion from a benign pituitary adenoma (>95% of cases) ^(1)^. Acromegaly develops slowly; thus, its diagnosis is often delayed ^(2)^. We report a case of acromegaly initially presenting with severe infectious diseases.

## Case Report

**Figure 1.**
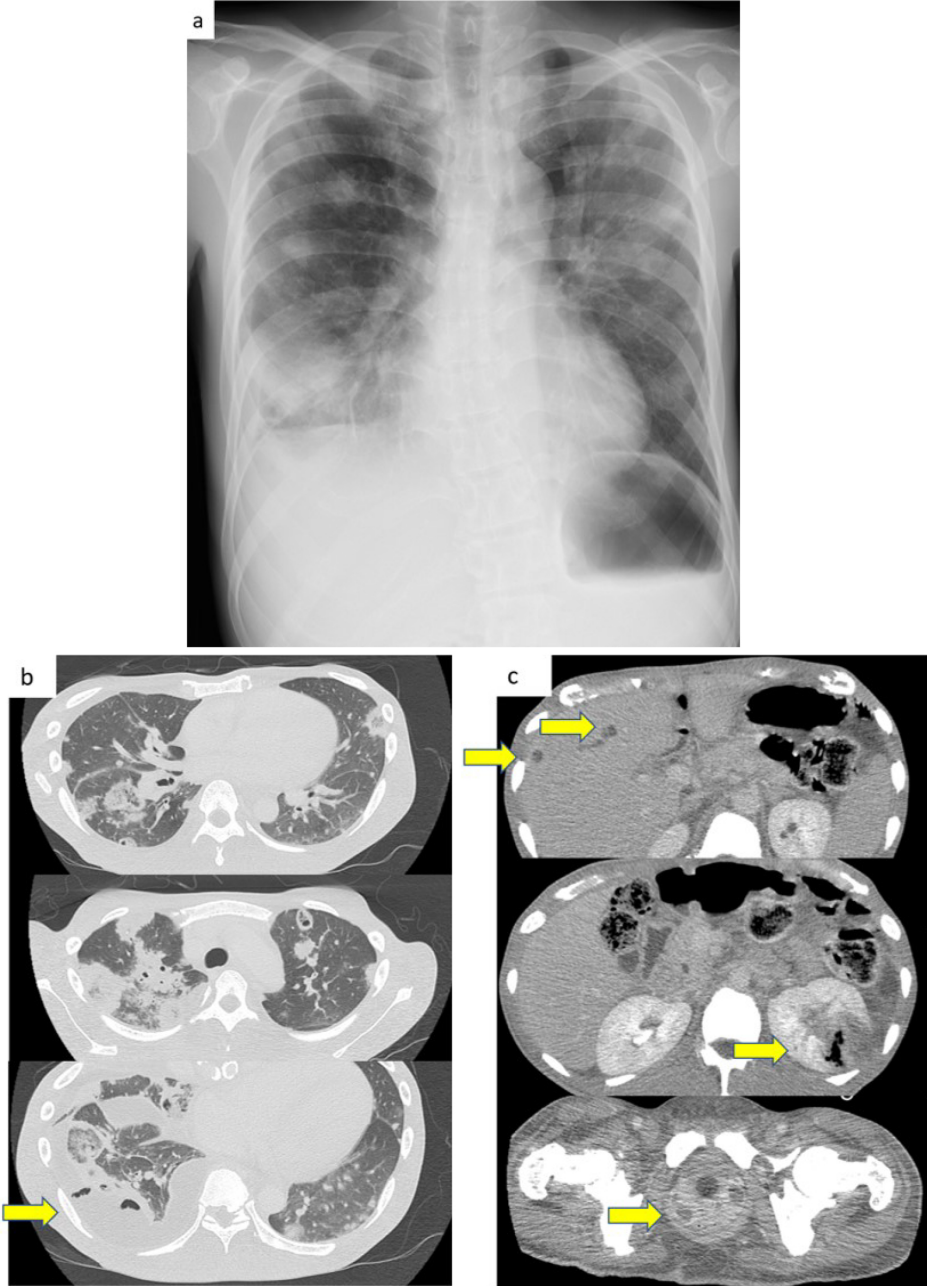
Imaging findings Figure 1-a. Chest radiograph at presentation shows multiple infiltrative shadows involving both lung fields Figure 1-b. Chest computed tomography performed on day 2 of hospitalization shows right empyema (arrow) Figure 1-c. Contrast-enhanced abdominal computed tomography shows liver abscess, emphysematous pyelonephritis, and a prostate abscess (arrow).

A 39-year-old man presented to our hospital with a 10 day history of worsening fever, cough, and fatigue. He was 176.0 cm tall and weighed 63.7 kg. His vital signs at the time of presentation were as follows: blood pressure, 149/97 mmHg; pulse rate, 111 beats/min; temperature, 37.2°C; and saturation at room air, 92%. Physical examinations at presentation revealed the following: chest auscultation, clear; cardiac sounds, no murmur. Chest radiography and chest computed tomography (CT) revealed multiple infiltrates in both lung fields (Figure 1-a and 1-b). Thus, he was admitted into the intensive care unit (ICU).

Table 1 shows that at the time of admission, blood tests revealed features suggestive of an increased inflammatory response and coagulation system, diabetic ketoacidosis (DKA), and sepsis. Those laboratory data and image findings indicated septic pulmonary embolism (SPE). Immediately after he was admitted into the ICU, oxygen supplementation, empiric antibiotics, and insulin therapy were initiated. On day 2 of hospitalization, additional contrast-enhanced abdominal CT scans showed exacerbation of multiple infiltrates in both lung fields, an appearance of right empyema, liver abscess, pyelonephritis, and a prostate abscess (Figure 1-c). *Klebsiella pneumoniae* was later identified on various cultures (Table 1), and the patient was switched to sensitive Cefotiam on day 6. As the patient’s condition improved, oxygen supplementation, intravenous antibiotics, and insulin were discontinued in sequence.

**Table 1.** Laboratory Findings on Admission (day-1).

Laboratory findings			
Blood test		Blood sugar	
White blood cell (10^3^/μL)	30.500	Random blood sugar (mg/dL)	572
Hemoglobin (g/dL)	13	Hemoglobin A_1c_ (%)	16.7
Platelet (10^4^/μL)	55.9	Coagulation test
Biochemical test		Prothrombin time (s)	11.8
Total protein (g/dL)	7.5	Activated partial thromboplastin time (sec)	31.3
Albumin (g/dL)	3.1	D-Dimer (μg/mL)	6.9
Aspartate aminotransferase (IU/L)	15	Urinalysis	
Alanine transaminase (IU/L)	30	PH	5.5
γ-GTP (IU/L)	28	Protein	+
Total bilirubin	0/5	Sugar	4+
Urea nitrogen (mg/dL)	19.9	Keton body	4+
Creatinine (mg/dL)	0.46	Occult body	2+
Na (mEq/L)	126	Nitrite	-
K (mEq/L)	4.1	Bacteria (/μL)	462
Cl (mEq/L)	90	White blood cell (/μL)	1228
Ca (mEq/L)	9.1	Culture	
C-reactive protein (mg/dL)	26.12	Sputum	*Klebsiella* *pneumoniae*
Procalcitonin (ng/mL)	2.94	Venous blood	*Klebsiella* *pneumoniae*
		Urine	*Klebsiella* *pneumoniae*
		Pleural effusion	*Klebsiella* *pneumoniae*

**Figure 2.**
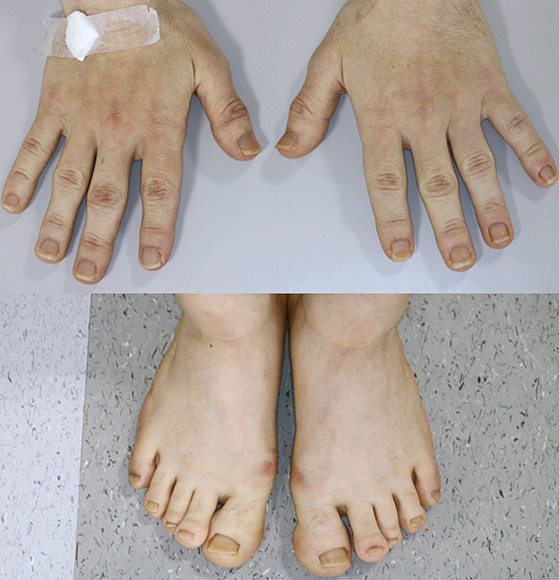
Physical characteristics of the patient: Increased sizes of both limbs.

While in the ICU, the ward nurse noticed that the patient’s toe tips were too large to fit the clamp device of a pulse oximeter; thus, we suspected acromegaly. When the patient improved, we re-examined his whole body and confirmed that he had clinical features indicative of acromegaly including bulging eyebrows, enlarged nose and lips, large feet, and prognathism (Figure 2). However, he and his family had not noticed these features, except for his enlarged feet. Moreover, we confirmed that the patient did not have a history of excessive consumption of sugar-containing soft drinks.

**Figure 3.**
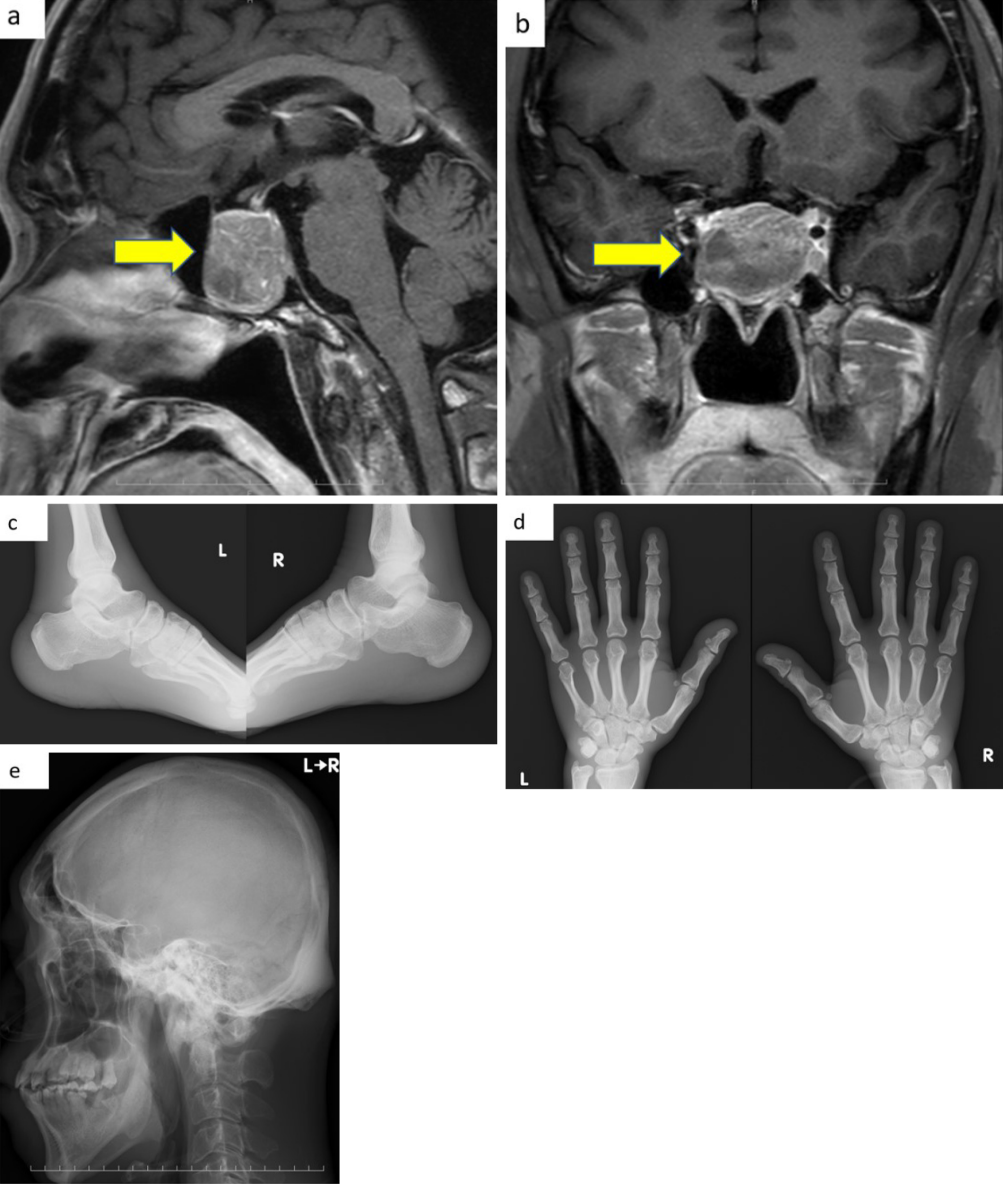
Contrast-enhanced head magnetic resonance image (T1-weighted image) shows a pituitary mass (arrow). (a) Sagittal section. (b) Coronal section Plain radiograph showing (c) heel pads (R 23 mm, L 25 mm); (d) cauliflower deformity; and (e) frontal sinus enlargement, sella turcica enlargement, mandibular protrusion, and malocclusion.

Figure 3-a and 3-b show that a pituitary mass was detected on contrast-enhanced head MRI on day 28. X-ray images revealed thickened heel pads, cauliflower deformity, frontal sinus enlargement, sella turcica enlargement, and mandibular malocclusion (Figure 3-c, 3-d and 3-e). Table 2 shows that a 75 g oral glucose tolerance test (75 g) on day 17 confirmed abnormal secretion of GH as it showed a paradoxical increase. Nevertheless, the blood insulin-like growth factor I (IGF-I) level was within the reference range on day 19. According to the clinical guidelines of the Japan Endocrine Society, physical and laboratory findings in this patient, other than his blood IGF-I level, met the diagnostic criteria for acromegaly. Despite blood IGF-I levels being normal, the patient was categorized as having definitive acromegaly because of uncontrolled diabetes mellitus, as stated in precautionary statement 3 of the guideline. Therefore, we diagnosed the patient with acromegaly, and he was referred to a specialized center for the treatment of acromegaly.

**Table 2.** Endocrine Test.

75 g oral glucose tolerance test (min) on day 17	Base line	30	60	90	120	150	180
Growth hormone (ng/mL)	317	447	458	454	870	815	820
Blood Glucose (mg/mL)	119	164	216	288	297	278	239
Insulin-like growth factor-I (ng/mL) on day 19	130						

Reference value of growth hormone: 0.13-9.88 ng/mL. Reference value of insulin-like growth factor-I: 95-266 ng/mL.

## Discussion

This patient was admitted with DKA, SPE, and severe infection but was diagnosed with acromegaly after a ward nurse noticed his enlarged toe tips. However, not only did this patient display clinical features indicative of acromegaly, but he also demonstrated DKA, which is another potential atypical feature of acromegaly and is rare in young adults. Particularly in Japan, where DKA is not common, DKA in younger to middle-aged adults may be a good indicator of acromegaly, as shown in a previous study ^(2)^. Additionally, although this is not a habit of this patient, excessive ingestion of sugar-containing soft drinks have been reported to promote DKA in patients with acromegaly ^(2)^. This must be recognized to establish an early diagnosis.

Another study also demonstrated a paradoxical increase in GH secretion by 75 g OGTT, which occurred in 10% of acromegaly patients ^(3)^, similar to this patient. In such patients, their daily diet may cause repeated increases in GH, which could accelerate the development of DKA through increased insulin resistance and lipolysis, similar to our hypothesis described in a previous study ^(2)^. Besides DKA, hirsutism ^(4)^; epilepsy ^(5)^; and wheezing, hemoptysis, dyspnea, or pneumonia ^(6)^ have been reported as atypical initial findings for acromegaly.

The clinical features of acromegaly develop slowly; thus, its diagnosis is often delayed ^(7)^. This patient and his family did not notice the various physical changes except for his enlarged feet. Acromegaly is diagnosed very late in a substantial number of patients, often when the disease is already in advanced stages ^(8)^.

Although diagnostic procedures for acromegaly have improved, such as using IGF-I as a simple diagnostic test, the diagnostic delay (DD) and the age at diagnosis have not improved ^(9), (10), (11)^ with a DD that ranges from 5 to 14 years ^(10), (11)^. Esposito et al. ^(12)^ reported that in a total of 603 patients with acromegaly, mean (S.D.), the DD was 5.5 (6.2) years [median (minimum, maximum) 3.3 (0.0-25.9)]. Furthermore, they indicated that prolonged DD is associated with increased morbidity and mortality.

Previous studies ^(13), (14)^ have shown that 39% of patients are diagnosed by internists or family physicians, and 36% by other specialists other than endocrinologists. Therefore, as seen in this case, other specialists or physicians, other than endocrinologists, should include acromegaly in the differential as soon as possible to ensure a better prognosis for patients. As described by Boguszewski ^(15)^, the motto “you must know it to think of it” is advocated in awareness efforts to reduce the time to diagnosis, and this will result in lower rates of morbidity and mortality.

We obtained written informed consent from the patient to publish the information including laboratory data, image finding, and his anonymous photos. Furthermore, this case report was approved by the institutional review board of the Ishikiriseiki Hospital on August 14, 2021 (approval number: 21-25).

## Article Information

### Conflicts of Interest

None

### Author Contributions

ET, DA, YO, YS, KH, NY, MN, and KM: acquisition of data, analysis, and interpretation of data and drafting of the manuscript; TH, MN, and YT: acquisition of data, analysis, and interpretation of data; and ET, TH, and, KM: design of the study and analysis and revision of the manuscript.

IRB Approval code and the Name of the institution that granted the approval: 21-25　Ishikiri-Seiki Hospital

This case report was conducted in accordance with the 1964 declaration of Helsinki and its later amendments.

## References

[1] Katznelson L, Laws Jr. ER, Melmed S, et al. Acromegaly: an endocrine society clinical practice guideline. J Clin Endocrinol Metab. 2014;99(11):3933-51. 25356808 10.1210/jc.2014-2700

[2] Yoshida N, Goto H, Suzuki H, et al. Ketoacidosis as the initial clinical condition in nine patients with acromegaly: a review of 860 cases at a single institute. Eur J Endocrinol. 2013;169(1):127-32. 23828957 10.1530/eje-13-0060

[3] Shlomo M. Acromegaly. N Engl J Med. 1990;322(14):966-77. 2179724 10.1056/NEJM199004053221405

[4] Rajesh J, Deep D, Shivaprasad KS, et al. Acromegaly presenting as hirsuitism: uncommon sinister aetiology of a common clinical sign. Indian J Endocrinol Metab. 2012;16(Suppl 2):S297. 23565405 10.4103/2230-8210.104066 PMC3603053

[5] Nyasatu GC, Ahlam AA, Abid MS, et al. Status epilepticus and diabetes ketoacidosis: uncommon clinical presentations of acromegaly. Endocrinol Diabetes Metab Case Rep. 2021;2021(1):1-6. 10.1530/EDM-20-0156 PMC8185527 33960324

[6] Odell DD, Michaud G, Alazemi S, et al. Acromegaly: an unusual presentation of bronchial carcinoid. Chest. 2009;136(4):41S.

[7] Melmed S. Medical progress: acromegaly. N Engl J Med. 2006;355(24):2558-73. 17167139 10.1056/NEJMra062453

[8] Colao A, Grasso LFS, Giustina A, et al. Acromegaly. Nat Rev Dis Primers. 2019;5(1):20. 30899019 10.1038/s41572-019-0071-6

[9] Reid TJ, Post KD, Bruce JN, et al. Features at diagnosis of 324 patients with acromegaly did not change from 1981 to 2006: acromegaly remains under-recognized and under-diagnosed. Clin Endocrinol. 2010;72(2):203-8. 10.1111/j.1365-2265.2009.03626.x PMC2866138 19473180

[10] Arosio M, Reimondo G, Malchiodi E, et al. Predictors of morbidity and mortality in acromegaly: an Italian survey. Eur J Endocrinol. 2012;167(2):189-98. 22596288 10.1530/EJE-12-0084

[11] Maione L, Chanson P. National acromegaly registries. Best Pract Res Clin Endocrinol Metab. 2019;33(2):101264. 30894298 10.1016/j.beem.2019.02.001

[12] Esposito D, Ragnarsson O, Johannsson G, et al. Prolonged diagnostic delay in acromegaly is associated with increased morbidity and mortality. Eur J Endocrinol. 2020;182(6):523-31. 32213651 10.1530/EJE-20-0019

[13] Drange MR, Fram NR, Herman-Bonert V, et al. Pituitary tumor registry: a novel clinical resource. J Clin Endocrinol Metab. 2000;85(1):168-74. 10634382 10.1210/jcem.85.1.6309

[14] Cordero RA, Barkan AL. Current diagnosis of acromegaly. Rev Endocr Metab Disord. 2008;9(1):13-9. 18236162 10.1007/s11154-007-9060-2

[15] Boguszewski CL. Acromegaly: ‘You must know it to think of it’. Eur J Endocrinol. 2020;183(1):C1-4. 32487776 10.1530/EJE-20-0281

